# ViSQA: A benchmark dataset and baseline models for Vietnamese spoken question answering

**DOI:** 10.1371/journal.pone.0340771

**Published:** 2026-01-12

**Authors:** Le Trong Minh, Nguyen Duc Thinh, Nguyen Khanh Tho Loc, Le Van Quan, Ngo Duc Tam, Le Hoang Son

**Affiliations:** 1 VNU University of Engineering and Technology, Vietnam National University, Hanoi, Vietnam; 2 Artificial Intelligence Research Center, VNU Information Technology Institute, Vietnam National University, Hanoi, Vietnam; 3 Faculty of Computer Science and Engineering, Thuyloi University, Hanoi, Vietnam; National University of Malaysia Faculty of Education: Universiti Kebangsaan Malaysia Fakulti Pendidikan, MALAYSIA

## Abstract

Spoken Question Answering (SQA) extends machine reading comprehension to spoken content and requires models to handle both automatic speech recognition (ASR) errors and downstream language understanding. Although large-scale SQA benchmarks exist for high-resource languages, Vietnamese remains underexplored due to the lack of standardized datasets. This paper introduces ViSQA, the first benchmark for Vietnamese Spoken Question Answering. ViSQA extends the UIT-ViQuAD corpus using a reproducible text-to-speech and ASR pipeline, resulting in over 13,000 question–answer pairs aligned with spoken inputs. The dataset includes clean and noise-degraded audio variants to enable systematic evaluation under varying transcription quality. Experiments with five transformer-based models show that ASR errors substantially degrade performance (e.g., ViT5 EM: 62.04% → 36.30%), while training on spoken transcriptions improves robustness (ViT5 EM: 36.30% → 50.70%). ViSQA provides a rigorous benchmark for evaluating Vietnamese SQA systems and enables systematic analysis of the impact of ASR errors on downstream reasoning.

## Introduction

Spoken Question Answering (SQA) has emerged as a critical task at the intersection of speech recognition and machine reading comprehension (MRC), driven by the rise of voice-based interfaces and virtual assistants. Unlike conventional text-based Question Answering (QA), where systems operate on clean textual passages, SQA requires models to comprehend and extract answers directly from spoken content, typically in the form of ASR-generated transcripts. This additional modality introduces unique challenges: models must cope with transcription inaccuracies, disfluencies, and the subtle characteristics of spoken language that differ significantly from carefully edited text.

In response to these challenges, several large-scale benchmark datasets have been developed for languages such as English and Chinese. Notably, the Spoken SQuAD [1] corpus extends the widely used SQuAD benchmark by converting text passages to speech via text-to-speech (TTS) synthesis and subsequently generating Automated Speech Recognition (ASR) transcripts, thereby enabling systematic evaluations of QA performance under realistic speech recognition conditions. Similarly, the ODSQA [2] dataset provides an open-domain spoken QA resource for Chinese, facilitating cross-lingual comparisons and methodological advancements in SQA.

However, the Vietnamese language, which is spoken by over 98 million people worldwide, has remained underrepresented in this area. While Vietnamese QA research has seen considerable progress in the textual domain, there has been a conspicuous absence of dedicated resources targeting the spoken modality. Several high-quality Vietnamese MRC datasets have emerged, including the general-domain UIT-ViQuAD [3], which contains over 23,000 QA pairs derived from Vietnamese Wikipedia articles, or the UIT-ViNewsQA [4], which focuses on healthcare information with more than 22,000 QA pairs curated from online health news. These benchmarks have significantly advanced the state of Vietnamese text-based QA. Nevertheless, they do not address the practical scenario in which users interact with systems through spoken queries about spoken or audio content, which remains a critical limitation in the era of ubiquitous voice assistants and conversational AI. This clear gap highlights the urgent need for a robust benchmark to systematically evaluate end-to-end Vietnamese SQA systems and to investigate how ASR imperfections affect downstream QA accuracy in this language context. Without such a resource, the development of reliable, voice-enabled question answering systems for Vietnamese remains constrained, limiting progress in both academic research and real-world deployment.

To bridge this gap, **this paper introduces ViSQA, the first Vietnamese Spoken Question Answering (SQA) dataset**. ViSQA extends the widely used UIT-ViQuAD corpus by synthesizing spoken contexts via high-quality TTS and generating corresponding ASR transcripts, yielding over 13,000 question-answer pairs aligned with realistic spoken inputs. The adoption of a fully synthetic TTS–ASR generation pipeline was a strategic design choice aimed at ensuring perfect reproducibility, allowing systematic control over transcription quality, and isolating the influence of ASR errors from confounding factors such as speaker variability or spontaneous disfluency. Such experimental control is essential for building a diagnostic benchmark that can quantify how different levels of recognition accuracy affect downstream reasoning performance. This mirrors the design philosophy of earlier English and Chinese SQA benchmarks, such as Spoken SQuAD [1] and ODSQA [2], where synthetic pipelines provided scalable and transparent conditions for early-stage research before moving to more natural speech. ViSQA extends this paradigm to Vietnamese, establishing a reproducible and extensible foundation on which subsequent, human-recorded extensions can be grounded and meaningfully interpreted.

To reflect diverse real-world acoustic conditions, ViSQA further provides augmented test sets incorporating controlled noise conditions and varying ASR error levels, enabling researchers to systematically evaluate model robustness and analyze sensitivity to ASR quality. Empirical benchmarks with state-of-the-art MRC models fine-tuned on ViSQA demonstrate how ASR quality can substantially impact answer extraction performance, underscoring the critical interplay between speech recognition and downstream language understanding in Vietnamese. By making ViSQA publicly available, this study aims to provide the research community with a standardized benchmark that will advance Vietnamese spoken-language processing. This work lays a foundational framework for more in-depth research on spoken question answering in Vietnam in the future.

**The contributions of this research** are summarized below:

- **Vietnamese SQA Dataset**: A dataset named ViSQA, one of the pioneering spoken question answering corpora for Vietnamese, is presented. An extended test set with higher noise and ASR errors is also provided to stress-test model robustness.
- **Dataset Creation Pipeline**: A reproducible TTS-ASR pipeline for generating realistic Vietnamese SQA data (WER: 6–15%) is introduced.
- **Extensive Experiments**: A comprehensive evaluation of state-of-the-art Vietnamese Machine Reading Comprehension (MRC) models is conducted to assess their performance on both the spoken question answering dataset and text-based dataset.

## Related works

Spoken Question Answering has gained considerable traction in recent years, with a growing body of research dedicated to enhancing the robustness, efficiency, and scalability of SQA systems. SQA typically involves the integration of multiple components, ranging from automatic speech recognition to question understanding and answer extraction. Consequently, various methods have been proposed to address the unique challenges of this task, including handling ASR errors, supporting multimodal inputs, and adapting to low-resource languages.

Studies of Menevşe *et al*. [5], Junjie Hu *et al*. [6] are among the pioneering works that proposed an automatic SQA system framework consisting of three steps: Question Generation, Text-To-Speech, and Automatic Speech Recognition, with the strength of flexibility when applied to low-resource languages through multilingual Transformer models. Additionally, several datasets and related studies have contributed to the development of this field. For instance, Spoken-CoQA (Chenyu You *et al*.) [7] focuses on building a multi-turn conversational QA dataset with over 40,000 QA pairs, and proposes the DDNet model, which employs knowledge distillation and dual attention mechanisms to exploit conversational context. This is a major step towards modeling spoken dialogues. From a different perspective, HeySQuAD (Yijing Wu *et al*.) [8] addresses the problem from a training data angle, showing that training models on speech data can significantly improve performance. This study highlights the critical role of constructing and leveraging real spoken data instead of relying solely on textual data. More recently, LibriSQA (Zhao *et al*.) [9] takes an end-to-end SQA system approach that eliminates the need for a separate ASR module. With a dataset of 214,000 QA pairs, the WavLM model demonstrates that jointly training ASR and QA can be mutually beneficial, reducing transmission errors and enhancing the overall smoothness of the SQA pipeline. These works collectively illustrate a progression from pipeline designs toward end-to-end models, while also revealing the data-intensive nature of such approaches. Additionally, a common pattern across these benchmarks is that early-stage spoken QA research often begins from controlled or synthetic audio rather than fully in-the-wild human speech. Spoken SQuAD and HeySQuAD, for example, deliberately rely on text-to-speech or re-synthesized material to generate large amounts of data with known content and controllable noise, thereby enabling systematic analysis of how ASR errors impact comprehension.

In the context of Vietnam, research on SQA remains limited. Most works focus on two separate directions: automatic question answering and ASR, without a clear integration between them to form a complete SQA system. On the QA side, ViNewsQA (Kiet *et al*.)[4] is a recent dataset designed for evaluating machine reading comprehension in Vietnamese, centered on online health articles with answers directly extracted from the text; VIMQA (Khang *et al*.) [10] is one of the standard Vietnamese datasets providing over 10,000 QA pairs based on Wikipedia articles; while UIT-ViQuAD (Kiet *et al*.) [3] expands toward multi-context QA, allowing evaluation of language understanding in complex paragraphs. However, these datasets are limited to clean text inputs and do not address speech-related challenges. More recently, the VLogQA (Thinh *et al*.) [11] study represents an interesting advancement by leveraging Vietnamese YouTube Vlog data, performing processing steps including dialogue segmentation, noise reduction, speech-to-text conversion, and question–answer annotation. On the ASR side, prior works such as VIVOS (Vu Hoang *et al*.) [12] and the VLSP ASR Challenge (Thanh *et al*.) [13] have laid the foundation for Vietnamese speech recognition by releasing large-scale datasets and building baseline models using architectures like Wav2Vec 2.0 and Conformer. These resources have enabled the development of more recent Vietnamese-specific ASR models, such as PhoWhisper (Thanh *et al*.) [14] or LingWav2Vec2 (Tuan *et al*.) [15].

Despite these advancements, a clear gap remains: there is currently no dedicated Vietnamese SQA dataset for jointly evaluating speech recognition and downstream QA performance. Existing Vietnamese QA datasets focus solely on clean text, while ASR datasets emphasize transcription accuracy without linking to higher-level language understanding tasks. This lack of integration limits the systematic study of how ASR quality affects spoken QA, hindering the development of robust, voice-enabled QA systems for Vietnamese. ViSQA is designed to fill precisely this gap. Following the proven methodological progression observed in international SQA benchmarks, the initial release of ViSQA adopts a synthetic, reproducible construction of TTS–ASR. This controlled design enables fine-grained investigation of error propagation before incorporating diverse human speech in future extensions. In this way, ViSQA provides both a much-needed Vietnamese SQA resource and the essential first layer of a longer-term research roadmap toward natural, real-world spoken question answering.

## Data creation pipeline

**Fig 1:**
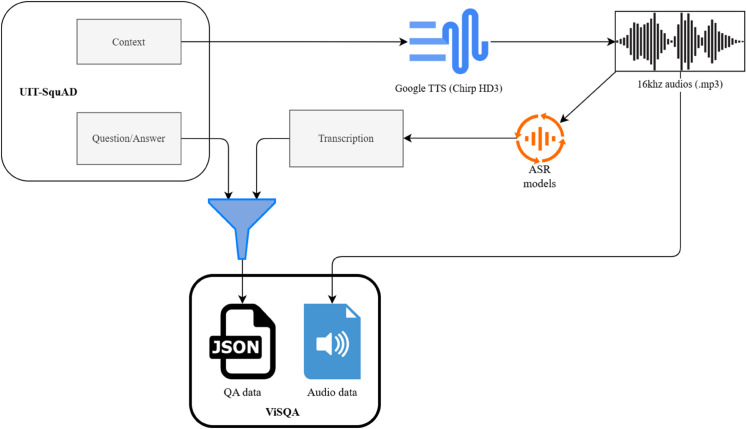
Data construction pipeline for the ViSQA dataset.

The ViSQA dataset was constructed by transforming the UIT-ViQuAD reading comprehension dataset, which was originally designed for Vietnamese text-based question answering, into a spoken question answering resource. UIT-ViQuAD consists of Vietnamese Wikipedia passages accompanied by curated questions, with each answer appearing as a text span within its corresponding context. Fig 1 illustrates the overall data construction pipeline for the ViSQA dataset. To simulate spoken interaction, the textual contexts were first converted into audio using the Google Cloud Text-to-Speech (Chirp) API. This system supports multiple Vietnamese voices, including variations in pitch and speed, allowing for more natural and diverse acoustic delivery. The resulting audio files were saved in .mp3 format at a 16kHz sampling rate and subsequently transcribed using the Google Cloud Speech-to-Text API. This process intentionally introduced realistic recognition errors, thereby reflecting the practical challenges of automatic speech recognition for Vietnamese.

The data construction process employs a TTS–ASR pipeline designed to ensure reproducibility and to isolate the effect of ASR errors under fully controlled conditions. By generating and re-transcribing speech with known parameters, the setup eliminates uncontrolled factors such as accent variation, spontaneous disfluency, or recording inconsistencies, which could otherwise confound analysis. This design enables a reproducible benchmark in which model differences can be attributed specifically to transcription noise. Comparable controlled or synthetic pipelines have been employed in benchmark-oriented studies [5, 7], demonstrating that reproducible setups for spoken QA can be effectively established before scaling toward natural human speech. In the same spirit, the synthetic construction of ViSQA is not a mere workaround but a methodological commitment: it turns the dataset into a well-defined diagnostic testbed for quantifying error propagation and evaluating robustness strategies under transparent, reproducible conditions.

Since the recognition errors errors could lead to the loss, corruption, or shift of the original answer span, additional preprocessing steps were required to ensure answerability. Each QA pair was subsequently processed to re-align the original textual answer with the ASR-generated transcript using a two-stage string-matching procedure. Candidate spans were first located through direct substring matches, including a normalized form where redundant whitespace was collapsed. If no direct match was found, the algorithm mapped the normalized position back to the original transcript and searched within a fixed window of ±50 characters around the estimated location. A span was accepted as a “confident” match if the original answer text was recovered exactly within that window; otherwise, the pair was discarded. In cases where plausible answers were defined in the original dataset, these were treated equivalently to the gold answer during matching. Furthermore, metadata was maintained to track whether the matched span originated from the original answer or from a plausible alternative, ensuring that even imperfect but valid matches could contribute to model training while still reflecting real-world uncertainty. Through this filtering and re-alignment mechanism, the integrity and utility of each QA pair was preserved. Since the test set of UIT-ViQuAD does not provide ground-truth answers, the validation set was used instead as the base for generating the ViSQA dataset, thereby ensuring that all examples had accessible gold and plausible answers necessary for the realignment and filtering procedures described above. The full re-alignment procedure is detailed in Algorithm 1.

**Algorithm 1 Align answer spans to ASR transcription.**

**Require:** Answer texts $\text{A}=\{{\text{a}}_{1},{\text{a}}_{2},\ldots ,{\text{a}}_{\text{n}}\}$, original start positions $\text{S}=\{{\text{s}}_{1},{\text{s}}_{2},\ldots ,{\text{s}}_{\text{n}}\}$, ASR transcript *T*

**Ensure:** Updated start positions ${\text{S}}^{\prime }=\{{\text{s}}_{1}^{\prime },{\text{s}}_{2}^{\prime },\ldots ,{\text{s}}_{\text{m}}^{\prime }\}$ for matched answers

1: Initialize ${\text{A}}^{\prime }\leftarrow [\;]$, ${\text{S}}^{\prime }\leftarrow [\;]$

2: **for** i←1 to *n* **do**

3:   $\text{a}\leftarrow {\text{a}}_{\text{i}}$, $\text{s}\leftarrow {\text{s}}_{\text{i}}$

4:   P← FINDEXACTMATCHES(*a*, *T*)

5:   **if** P=∅ **then**

6:    ${\text{a}}_{\text{norm}}\leftarrow$ NORMALIZE(*a*)

7:    ${\text{T}}_{\text{norm}}\leftarrow$ NORMALIZE(*T*)

8:    ${\text{P}}_{\text{norm}}\leftarrow$ FINDEXACTMATCHES(${\text{a}}_{\text{norm}}$, ${\text{T}}_{\text{norm}}$)

9:    **for all** ${\text{p}}_{\text{norm}}\in {\text{P}}_{\text{norm}}$ **do**

10:     Estimate p← MAPTOORIGINAL(${\text{p}}_{\text{norm}}$, *T*, ${\text{T}}_{\text{norm}}$)

11:     $p^{\prime }\leftarrow$ SEARCHNEARBY(*T*, *a*, *p*)

12:     **if** ${\text{p}}^{\prime }$ found **then**

13:      $\text{P}\leftarrow \text{P}\cup \{{\text{p}}^{\prime }\}$

14:     **else**

15:      P←P∪{p}

16:     **end if**

17:    **end for**

18:   **end if**

19:   **if** P≠∅ **then**

20:    ${\text{s}}^{\prime }\leftarrow$ CLOSESTTO(*s*, *P*)

21:    Append *a* to ${\text{A}}^{\prime }$, Append ${\text{s}}^{\prime }$ to ${\text{S}}^{\prime }$

22:   **end if**

23: **end for**

24: textbfreturn ${\text{A}}^{\prime },{\text{S}}^{\prime }$

**Notes.** FindExactMatches: returns start indices of exact matches. MapToOriginal: projects normalized index back to original text. SearchNearby: scans within a ±50 character window around an estimated position. ClosestTo: selects candidate minimizing |p−s| relative to original start *s*.

To assess the quality of ASR transcriptions on clean audio, the Word Error Rate (WER) metric was calculated for the training and validation splits generated by Google Speech-to-Text. As shown in Table 1, although the transcriptions are generally intelligible, recognition errors still occur frequently enough to distort answer spans, highlighting the need for careful preprocessing and alignment when constructing spoken QA datasets. To increase the realism and difficulty of the evaluation, background noise was also introduced into the test set. Two noisy variants were produced by combining synthetic audio effects, such as reverb, bandpass filtering, or clipping distortion using torch-audiomentations, with real-world environmental sounds from the ESC-50 dataset, which contains street noise, animal sounds, and household activities. This noise augmentation strategy simulates moderate to high-noise conditions and offers a more practical test scenario than using only artificial white noise. Table 2 presents the impact of background noise on ASR transcription accuracy. By providing the same set of QA pairs under different noise conditions, researchers can systematically vary the transcription quality and observe the downstream impact on question-answering model performance. In this way, ViSQA functions as a reproducible diagnostic testbed that allows detailed tracing of how changes in word-error rate, insertion or deletion errors, and noise levels propagate through to comprehension metrics. This capability enables benchmarking of robustness of SQA models under controlled error regimes, encourages the design of ASR-error-correction or transcript-repair modules targeted to preserve QA performance facilitates the exploration of adaptive SQA architectures that dynamically respond to varying transcription quality.

**Table 1:** Transcription accuracy on clean audio using Google Speech-to-Text.

ASR System	Train WER	Test WER
Google Speech-to-Text	9.60%	11.02%

**Table 2:** Word Error Rate (WER) under noisy conditions.

	No Noise	Noise
Testing Set	11.02%	15.83%

To provide a clearer understanding of the dataset, we present qualitative samples that illustrate how passages, questions, answers, and ASR transcripts are represented in ViSQA. Table 3 shows a representative data sample, including the original text passage, its corresponding question, the gold answer span annotated within the passage, the ASR transcript generated from clean audio with recognition errors highlighted in bold, the ASR transcript generated from noisy audio with errors similarly highlighted, and an indication of whether the gold answer span was successfully re-aligned under both conditions.

**Table 3:** A qualitative sample from the ViSQA dataset. The example shows the passage, question, gold answer, ASR transcripts from clean and noisy audio (with ASR errors highlighted in bold), and whether the gold span was successfully re-aligned.

Item	Content
Passage	paris có một lịch sử lâu đời ... vào thế kỷ một tới thế kỷ sáu vua clovis một lấy paris làm thủ đô cho vương quốc franc ... belle époque *(Paris has a long history... From the 1st century to the 6th century, King Clovis I established Paris as the capital of the Frankish kingdom... Belle Époque.)*
Question	Paris bị Đế quốc Roma chiếm đóng vào thời điểm nào? *(When was Paris occupied by the Roman Empire?)*
Gold Answer Span	Text: thế kỷ 1 (start: 170, end: 177) (*the 1st century*)
Clean ASR Transcript	paris có một lịch sử lâu đời ... trở thành một thành phố la mã vào thế kỷ một tới thế kỷ sáu vua **clovid a** lấy paris làm thủ đô cho vương quốc **frang** ... **bellypod** *(Paris has a long history... It became a Roman city in the 1st century. From the 1st century to the 6th century, King* ***Clovid a*** *made Paris the capital of the* ***frang*** *...* ***bellypod***)
Noisy ASR Transcript	**và** đây chính là lịch sử lâu đời ... trở thành một thành phố la mã vào thế kỷ một tới thế kỷ sáu vua **clovid a** lấy paris làm thủ đô cho vương quốc **frank** ... **bellerport** ... *(And this is its long history... It became a Roman city in the 1st century. From the 1st to the 6th century, King* ***Clovid a*** *made Paris the capital of the* ***frank*** *...* ***bellerpod****...)*
Re-alignment	Gold answer span was successfully re-aligned in both clean and noisy transcripts. New indices: start ≈ 166 (clean), start ≈ 166 (noisy).

The final dataset includes 10,441 QA pairs for training and 1,507 QA pairs in the clean test set, with an additional noisy test set derived from it to enable robustness testing. A comparison with the original UIT-ViQuAD dataset and other closely related datasets is provided in Table 4. Unlike UIT-ViQuAD and ViNewsQA, which remain limited to text-only inputs, ViSQA extends Vietnamese QA into the spoken domain by incorporating audio synthesis and controlled noise variants. While VlogQA also integrates spoken content, its focus lies in natural conversational scenarios within narrower domains such as food and travel. In contrast, ViSQA is purpose-built as an open-domain benchmark, explicitly designed to measure and analyze the effect of ASR errors and acoustic degradation on QA performance. This makes ViSQA not just a dataset but a critical testbed for advancing robust and error-tolerant SQA research in Vietnamese.

**Table 4:** Comparison of dataset characteristics across UIT-ViQuAD, ViNewsQA, VlogQA, and ViSQA.

Dataset	Modality	Domain	Train QA	Test QA
UIT-ViQuAD	Text	Open	18,579	2,210
ViNewsQA	Text	Medical + News	17,568	1,992
VlogQA	Text + Spoken	Food + Travel	8,047	1,012
ViSQA	Text + Spoken	**Open**	**10,441**	**1,507**

## Machine reading comprehension model

In this section, we experiment with five Transformer-based language models that represent two major paradigms in machine reading comprehension: encoder-only architectures (PhoBERT, mBERT, XLM-R), which excel at span extraction tasks, and encoder–decoder architectures (BARTPho, ViT5), which are better suited for generative QA. This choice enables us to analyze how different model families handle the challenges of Spoken QA, particularly under noisy ASR-transcribed inputs:

- **PhoBERT** [16], proposed by Dat Quoc Nguyen (2020), is a monolingual Vietnamese RoBERTa variant, trained from scratch on a large Vietnamese corpus. Its language-specific optimization allows superior representation of Vietnamese syntax and semantics, making it a strong baseline for text-based QA. However, its lack of multilingual exposure may reduce adaptability to code-switching or cross-lingual tasks.
- **mBERT** [17] (Jind řich *et al*., 2019) is a multilingual BERT model trained on Wikipedia data across 104 languages with a shared WordPiece vocabulary. It leverages parameter sharing to learn cross-lingual semantic representations. For Vietnamese SQA, this broader lexical coverage may mitigate some ASR-induced errors (e.g., when transcripts contain foreign or code-switched terms). However, its lack of explicit alignment across languages and smaller per-language capacity can reduce precision compared to monolingual models.
- **BARTPho** [18] (Luong *et al*., 2022) builds upon the BART architecture, a denoising autoencoder designed for sequence-to-sequence task, through additional pre-training on Vietnamese data using span masking and sentence permutation objectives. This enables the model to excel in Vietnamese text generation tasks such as text infilling and sentence rewriting.
- **ViT5** [19] (Long *et al*., 2022) is a Vietnamese adaptation of the T5 (Text-to-Text Transfer Transformer) model. In SQA, its span corruption and translation-based pre-training objectives help it adapt to disfluent and error-prone transcripts. ViT5 can generate fluent answers even when the ASR output is fragmented, which makes it a strong candidate for robust spoken QA.
- **XLM-R** [20] (Conneau *et al*., 2020) is a multilingual RoBERTa-based model trained on a massive 2.5TB CommonCrawl dataset covering 100 languages. By scaling both the training data and model size, XLM-R generates robust multilingual representations that transfer effectively to low-resource languages such as Vietnamese.

By including these five models, our framework systematically compares monolingual vs. multilingual designs and encoder-only vs. encoder–decoder architectures. This comparative setup is critical to determine which modeling choices best mitigate ASR-induced errors and support robust Vietnamese SQA systems.

**Fig 2:**
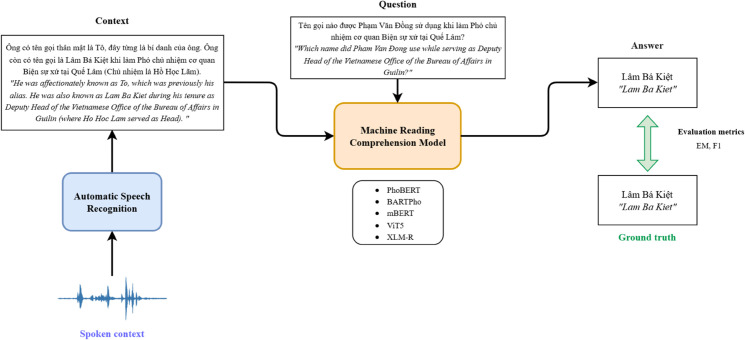
Overview diagram of the SQA framework.

Fig 2 illustrates the flow diagram of the Spoken Question Answering framework for Vietnamese. In this pipeline, during the testing phase, the spoken context data is first processed by an Automatic Speech Recognition system to generate the corresponding text transcription. This transcript, together with the input question, is then fed into a Machine Reading Comprehension model to extract the relevant answer span from the transcribed context. We first train the MRC models on the UIT-SQuAD training set and evaluate on both the UIT-SQuAD development set and the ViSQA test set to assess performance degradation when transitioning from clean text to ASR-transcribed input. Additionally, the models are retrained on the ViSQA training set and re-evaluated on the ViSQA test set to determine whether exposure to ASR data enhances model robustness, an essential trait for real-world spoken SQA applications.

## Experimental results

This section conducts a comprehensive evaluation of the Machine Comprehension Model for Vietnamese using the ViSQA dataset. The model’s performance is first assessed on both manually transcribed text and automatic speech recognition outputs. Subsequently, the impact of ASR errors on model performance is analyzed by conducting experiments with different ASR systems and by introducing varying levels of background noise. The evaluation is primarily conducted using two standard metrics: Exact Match (EM) and F1 score.

### Experiment setup

All experiments were conducted using two NVIDIA RTX 6000 GPUs, each equipped with 24 GB of VRAM. The models were fine-tuned for 10 epochs with a batch size of 16 and a maximum sequence length of 512 tokens. A learning rate of 4e-5 was used, and the AdamW optimizer was employed for parameter updates. To enhance training stability and convergence, a linear learning rate scheduler with warm-up was applied. This experimental configuration was consistently maintained across all model variants and evaluation scenarios to ensure a fair comparison.

### Results and discussions

Table 5 presents a detailed evaluation of five state-of-the-art language models to illustrate the impact of spoken input on question answering performance. All models were trained exclusively on the full UIT-ViQuAD training dataset, which contains only text-based Vietnamese question answering pairs. For evaluation, each model was tested on two distinct datasets: the UIT-ViQuAD-dev set and the ViSQA-test set. As shown in the table, all models exhibit a clear drop in both EM and F1 scores when evaluated on the spoken ViSQA-test set compared to the original UIT-ViQuAD-dev split, highlighting the significant impact of ASR errors and speech variability. ViT5 consistently achieves the highest scores across both conditions, indicating stronger robustness to noise, with EM and F1 dropping from 62.04% and 80.93% on UIT-ViQuAD-dev to 36.30% and 63.46% on ViSQA-test, respectively. PhoBERT shows similar resilience, though with a slightly larger performance gap. mBERT and XLM-R also maintain strong performance on clean text but suffer notable degradation under spoken conditions. Overall, the results confirm that models trained solely on text face considerable challenges when applied to spoken question answering without additional adaptation.

**Table 5:** Comprehensive evaluation of state-of-the-art models demonstrating performance degradation across spoken data conditions. All models were trained on the complete UIT-ViQuAD training dataset. The UIT-ViQuAD-dev and ViSQA-test represent the testing sets of UIT-ViQuAD and ViSQA, respectively.

Model	UIT-ViQuAD-dev		ViSQA-test	
	EM (%)	F1 (%)	EM (%)	F1 (%)
PhoBERT	58.05	76.46	31.85	53.32
ViT5	62.04	80.93	36.30	63.46
BartPho	49.30	71.77	23.42	53.17
mBERT	59.44	78.46	23.69	43.36
XLM-R	59.63	79.02	33.05	55.98

Subsequently, the aforementioned MRC models were trained on ViSQA’s spoken transcriptions. Table 6 presents a performance comparison between models trained on clean text documents (the UIT-SQuAD training set, referred to as Text) and those trained on ASR transcriptions (the ViSQA training set, referred to as Spoken). As shown in the table, all five models fine-tuned on ViSQA’s spoken transcriptions consistently outperform their counterparts trained on UIT-ViQuAD’s clean text when tested on the ViSQA-test set, which contains ASR-induced errors. For example, mBERT’s scores improve from 23.69 EM and 43.36 F1 (text training) to 46.18 EM and 69.09 F1 (spoken training), demonstrating a substantial gain in robustness. Similarly, XLM-R’s EM rises from 33.05 to 47.71, with its F1 score increasing from 55.98 to 70.25. These consistent improvements across models indicate that training directly on spoken transcriptions is an effective strategy for mitigating the impact of ASR errors in spoken question answering.

**Table 6:** Performance comparison of models on ViSQA-dev set. “Text” indicates models trained on clean text documents (UIT-ViQuAD), while “Spoken” refers to models trained on ASR transcriptions (ViSQA).

Model		ViSQA-test	
		EM (%)	F1 (%)
PhoBERT	Text	31.85	53.32
	Spoken	45.46	67.13
ViT5	Text	36.30	63.46
	Spoken	50.70	73.09
BartPho	Text	23.42	53.17
	Spoken	31.59	59.51
mBERT	Text	23.69	43.36
	Spoken	46.18	69.09
XLM-R	Text	33.05	55.98
	Spoken	47.71	70.25

To investigate the impact of noise on spoken question answering performance, Table 7 compares the results of language models evaluated on the same ViSQA test set under two conditions: clean input and input degraded with additional noise. All models were trained on the same ViSQA training set, ensuring that any performance gap reflects only their sensitivity to acoustic distortions. Notably, when the WER rises from 11.02% on the clean test set to 15.83% on the noise-added version, all models show a clear and uniform decline in both EM and F1 scores. Interestingly, the extent of this drop is similar across models and aligns closely with the moderate increase in WER, underscoring how even small degradations in transcription quality can steadily erode QA accuracy.

**Table 7:** Evaluation results of pre-trained language models on the ViSQA test set under clean and noisy conditions. All models were trained on the same ViSQA training set.

Model	No noise		Noise	
	EM (%)	F1 (%)	EM (%)	F1 (%)
PhoBERT	45.45	67.13	40.73	64.13
ViT5	50.70	73.09	45.65	70.56
BARTPho	31.59	59.51	28.47	56.74
mBERT	46.18	69.08	42.07	65.77
XLM-R	47.71	70.25	42.87	67.41

Furthermore, the AssemblyAI ASR API is utilized to generate higher-quality transcriptions of the ViSQA context compared to those produced by Google Speech-to-Text, which was employed in all preceding experiments. The word error rates (WER) of these two ASR systems on the ViSQA dataset are presented in Table 8. To examine the impact of different ASR systems on spoken question answering performance, a cross-evaluation is carried out in which MRC models are trained and tested under matched conditions using transcripts produced by the two aforementioned ASR engines. Table 9 reveals two key findings about the interaction between ASR quality and SQA accuracy. First, the choice of test transcripts dominates overall performance: switching from Google to higher-quality Assembly transcripts increases F1 by roughly 2–3 points for models trained on Google data (e.g., ViT5: 73.10 → 75.27; XLM-R: 70.25 → 72.68), confirming that downstream QA accuracy is tightly coupled to transcript fidelity. Second, training on the better ASR output further amplifies those gains and yields the most robust models. When both training and testing use Assembly transcripts, every model attains its peak scores. ViT5 reaches 57.80 EM, 77.08 F1, and even the weaker BartPho rises from 26.81 EM, 55.86 F1 (train with Assembly, test with Google) to 41.49 EM, 65.93 F1 (train with Assembly, test with Assembly), an absolute F1 increase of 10.07 points. Overall, the results emphasize that higher transcription quality at both training and evaluation stages tends to yield better QA performance. The discrepancies in exact match (EM) and F1 scores across different ASR settings suggest that ASR-induced errors, such as deletions, substitutions, or misalignments, significantly impact model effectiveness. Therefore, for practical SQA deployment, careful selection or enhancement of the ASR component can be as critical as model architecture or training strategy.

**Table 8:** WER of transcribed ViSQA context by Google STT and AssemblyAI.

ASR System	Train WER	Test WER
Google Speech-to-Text	9.60%	11.02%
AssemblyAI	6.11%	7.31%

**Table 9:** Performance comparison of machine comprehension models trained on Google vs. Assembly transcripts, evaluated under matched ASR conditions.

	Test ASR	Google		Assembly	
Train ASR	Model\Metric	EM (%)	F1 (%)	EM (%)	F1 (%)
Google	PhoBERT	45.46	67.13	46.29	67.71
	ViT5	50.70	73.10	54.31	75.27
	BartPho	31.59	59.51	35.55	61.03
	mBERT	46.18	69.09	51.96	72.50
	XLM-R	47.71	70.25	52.18	72.68
Assembly	PhoBERT	38.02	61.46	50.33	69.26
	ViT5	46.52	70.72	57.80	77.08
	BartPho	26.81	55.86	41.49	65.93
	mBERT	39.42	65.07	53.65	74.53
	XLM-R	42.47	66.70	55.18	74.64

Taken together, these experiments demonstrate how ViSQA functions in practice as a diagnostic testbed rather than only a static benchmark. By holding the linguistic content constant and systematically varying ASR source (Google versus Assembly), background noise, and training regime (text versus spoken transcripts), we can observe structured, interpretable patterns in EM/F1 degradation and recovery. For example, the consistent gains when switching to higher-quality Assembly transcripts, and the further improvements when training on those transcripts, show that ViSQA can disentangle the contributions of ASR quality and model adaptation. Similarly, the controlled WER increase from 11.02% to 15.83% and the corresponding, approximately proportional drop in QA scores illustrate how incremental acoustic degradation translates into measurable losses in comprehension. These analyses are only possible because the dataset’s synthetic construction decouples content from acoustic and ASR variability, allowing fine-grained probing of robustness strategies, error-correction modules, and end-to-end architectures within a stable experimental environment.

In order to mitigate the effects of stochastic initialization and optimization, each model was re-trained with five different random seeds. Table 10 reports the mean and standard deviation of EM and F1 scores across these runs. The relatively small standard deviations (all below 1.0) indicate that performance is stable across random restarts, suggesting that the observed differences between models primarily reflect genuine performance gaps rather than randomness. To further examine whether these gaps are statistically significant, we performed pairwise paired t-tests on the EM and F1 scores between every pair of models. The resulting p-values are summarized in Table 11. Each entry reports two p-values from paired t-tests on the EM and F1 scores, scaled by 10^−4^; for example, the entry “1.4;0.2” for PhoBERT vs. ViT5 corresponds to $p=1.4\;\times \;10^{-4}$ for EM and $p=0.2\;\times \;10^{-4}$ for F1. Cells marked “*NA*” indicate self-comparisons, which are not applicable. The majority of p-values are extremely small, and even the highest values remain low in absolute terms, confirming that the observed differences are statistically significant rather than arising from chance variation. This additional test provides statistical evidence that the observed differences are not attributable to chance but reflect genuine distinctions among the models.

**Table 10:** Mean and standard deviation of model performance on the *ViSQA-test* set, averaged over 5 re-training runs with different random seeds.

Model	ViSQA-test	
	EM (%)	F1 (%)
PhoBERT	44.45 ± 0.88	66.39 ± 0.60
ViT5	50.67 ± 0.56	72.95 ± 0.20
BARTPho	31.18 ± 0.40	59.46 ± 0.17
mBERT	45.57 ± 0.77	69.08 ± 0.19
XLM-R	47.34 ± 0.57	69.91 ± 0.42

**Table 11:** Pairwise p-values from paired t-tests on EM and F1 scores respectively between models (scaled by 10^−4^, rounded to 1 decimal).

Model	Phobert	ViT5	mBERT	BARTpho	XLM-R
Phobert	NA	1.4; 0.2	13.2; 3.0	0.0; 0.1	16.8; 1.1
ViT5	1.4; 0.2	NA	2.0; 0.0	0.0; 0.0	18.3; 0.9
mBERT	13.2; 3.0	2.0; 0.0	NA	0.0; 0.0	47.9; 149.7
BARTpho	0.0; 0.1	0.0; 0.0	0.0; 0.0	NA	0.0; 0.0
XLM-R	16.8; 1.1	18.3; 0.9	47.9; 149.7	0.0; 0.0	NA

Lastly, the ViSQA test set was categorized into distinct question types, as presented in Table 12 for the purpose of error analysis. Among these categories, “What” questions were the most frequent, while “Others” questions appeared least often. The number of correct answers and the corresponding accuracy for each question type were recorded in the table to enable a comparative evaluation across five pre-trained language models. Overall, the results indicate that no single model consistently outperforms the others across all question types. Instead, different models exhibit strengths in different categories. ViT5 emerges as the most competitive model, achieving the best accuracies on “What” (45.99%), “How” (50.90%), “Who” (50.70%), “Which” (59.60%), and “Why” (45.38%) questions, showing its robustness across both factoid and reasoning-oriented queries. XLM-R performs best on “When” questions with an accuracy of 69.48%, demonstrating strong temporal reasoning capabilities. PhoBERT achieves the highest performance on “Where” questions (47.22%), while mBERT is most effective in the “Others” category (66.67%) and remains competitive on “Where” and “Which” questions. By contrast, BARTPho consistently lags behind the other models, with particularly low performance on “Others” (13.33%) and “Who” (22.54%). A notable trend across all models is the continued difficulty with “Why” and “Others” question types, where accuracies remain significantly lower than other categories. These questions often require causal inference or answers that are less explicitly stated in the context, highlighting the persistent challenges of machine reading comprehension when moving beyond surface-level textual information.

**Table 12:** The count and accuracy rate of correct answers on the ViSQA test set, categorized by type, measured using the EM metric.

Question type	Total	BARTPho	mBERT	ViT5	PhoBERT	XLM-R
What	711	204	285	327	295	305
		0.2869	0.4008	0.4599	0.4149	0.4290
Where	72	30	40	32	34	33
		0.4167	0.5556	0.4444	0.4722	0.4583
When	154	82	104	103	97	107
		0.5325	0.6753	0.6688	0.6299	0.6948
How	167	45	76	85	76	71
		0.2695	0.4551	0.5090	0.4551	0.4251
Who	71	16	26	36	24	34
		0.2254	0.3662	0.5070	0.3380	0.4789
Which	198	66	110	118	107	111
		0.3333	0.5556	0.5960	0.5404	0.5606
Why	119	31	45	54	44	50
		0.2605	0.3782	0.4538	0.3697	0.4202
Others	15	2	10	9	8	8
		0.1333	0.6667	0.6000	0.5333	0.5333

## Conclusions

The development and introduction of ViSQA mark a significant advancement in addressing the underrepresentation of Vietnamese in Spoken Question Answering research. By providing the first publicly available dataset specifically tailored for Vietnamese SQA, this work bridges a critical gap, enabling systematic evaluation of how Automatic Speech Recognition quality impacts downstream question answering performance. Our experiments show that even moderate recognition errors substantially degrade accuracy, for example, ViT5 EM dropped from 62.04% with text data to 36.30% when paired with ASR transcripts (WER 11.02%), and performance declined further under noisier conditions (WER 15.83%). At the same time, training directly on ASR transcripts significantly improved robustness, as seen with mBERT EM increasing from 23.69% to 46.18%. These findings underscore the need for more resilient ASR systems and specialized datasets that jointly support the advancement of Vietnamese SQA. Beyond serving as a benchmark, ViSQA functions as a diagnostic testbed that facilitates fine-grained analysis of error propagation and model robustness. The controlled, synthetic design establishes an ideal foundation for researchers to quantify how specific changes in WER, error types, and noise conditions impact downstream reasoning, and to evaluate mitigation strategies under controlled settings. This design is the first and indispensable layer of a broader research roadmap. After creating a controlled synthetic baseline, we can scale toward natural human speech and analyze how the added variability influences spoken QA.

Collectively, this work not only establishes the foundational benchmark for Vietnamese SQA but also provides a scalable platform for advancing robust, inclusive, and practically deployable voice-based AI systems in Vietnamese-speaking environments. We hope that this combination of methodological clarity and extensibility will make ViSQA a useful tool for both developing robust SQA models and for understanding, in a scientifically principled manner, how speech recognition quality shapes question answering in Vietnamese.

## Future works

Building upon the diagnostic capabilities established in this study, several research avenues emerge as essential for advancing reliable Vietnamese Spoken Question Answering.

Firstly, our cross-evaluation experiments confirm that a modest reduction in transcription error rate yields disproportionately large downstream improvements, such as when switching from Google Speech-to-Text (WER 11.02%) to AssemblyAI (WER 7.31%), which increases ViT5 F1 from 73.10% to 77.08% on the same test set. This confirms that enhancing ASR fidelity, particularly through semantic-preserving error correction targeting entities, numbers, or temporal markers, will directly strengthen SQA robustness. At the same time, our results reveal that architectural choices fundamentally influence noise sensitivity: encoder–decoder models like ViT5 consistently outperform span-extraction approaches on ASR transcripts, indicating that answer generation with inherent denoising capability is better suited to reconstructing meaning under transcription fragmentation. Future work will therefore pursue an integrated strategy that couples improved ASR front-end quality with generative, denoising-aware MRC architectures, enabling the system to simultaneously reduce error introduction and increase resilience to the errors that remain.

Secondly, although exposure to ASR data substantially improves overall robustness, the persistent weakness observed in causal (“Why”) and inferential (“Others”) question types indicates that transcription robustness alone is insufficient to ensure semantic reliability. As shown in Table 12, accuracies for these categories remain markedly lower than for factoid questions across all assessed models. These question types require reasoning over implicit relations and maintaining logical coherence even when discourse markers or connective phrases are degraded by ASR. Future work must therefore explore mechanisms that stabilize and enrich high-level semantic representations, such as knowledge-grounded inference to compensate for missing textual evidence, or structured attention designs that are resilient to the omission of critical connective words.

Finally, our future work should extend this benchmark data by incorporating human-recorded samples such as crowdsourced or scripted dialogues from diverse Vietnamese speakers to quantify performance degradation arising from spontaneous speech phenomena including hesitations, regional accents, and code-switching. This expansion will bridge the synthetic-to-real gap and enable controlled ablation studies comparing synthetic and real speech conditions rather than ad hoc accumulation of heterogeneous data. Such methodological care is essential for creating a robust and inclusive SQA system capable of serving the full range of Vietnamese communication styles encountered in practical conversational settings.

In conclusion, these planned advancements establish a coherent long-term research agenda toward practical spoken-language AI for Vietnamese users. By leveraging ViSQA not only as a benchmark but also as a diagnostic instrument, future work can systematically target the core failure modes identified here and accelerate the development of SQA systems that remain accurate, robust, and inclusive in real-world acoustic environments.
